# Single Layer Centrifugation Improves the Quality of Fresh Donkey Semen and Modifies the Sperm Ability to Interact with Polymorphonuclear Neutrophils

**DOI:** 10.3390/ani10112128

**Published:** 2020-11-16

**Authors:** Marion Papas, Jaime Catalán, Sandra Recuero, Jane M. Morrell, Marc Yeste, Jordi Miró

**Affiliations:** 1 Equine Reproduction Service, Department of Animal Medicine and Surgery, Faculty of Veterinary Sciences, Autonomous University of Barcelona, E-08193 Bellaterra (Cerdanyola del Vallès), Spain; papas.marion@gmail.com (M.P.); dr.jcatalan@gmail.com (J.C.); 2 Biotechnology of Animal and Human Reproduction (TechnoSperm), Institute of Food and Agricultural Technology, University of Girona, E-17003 Girona, Spain; sandra.recuero@udg.edu; 3 Unit of Cell Biology, Department of Biology, Faculty of Sciences, University of Girona, E-17003 Girona, Spain; 4 Clinical Sciences, Swedish University of Agricultural Sciences, Box 7054, SE-75007 Uppsala, Sweden; jane.morrell@slu.se

**Keywords:** sperm, fresh semen, donkey, single layer centrifugation, sperm–PMN binding

## Abstract

**Simple Summary:**

Donkey Artificial Insemination (AI) with frozen/thawed semen results in poor fertility outcomes. Jennies show a significant post-AI endometrial reaction, with a large amount of defense cells—polymorphonuclear neutrophils (PMN)—migrating to the uterine lumen. Seminal plasma (SP) has a detrimental effect on sperm conservation and its removal is a necessary step in the semen freezing protocol. However, several SP proteins seem to control sperm-PMN binding. Single layer centrifugation (SLC) with colloids, which has been used to select spermatozoa and improve reproductive performance in different species, is known to remove SP proteins attached to the sperm membrane. In this study, two experiments were performed. The first one compared the quality of SLC-selected and non-selected fresh donkey spermatozoa. In the second experiment, PMN obtained from the peripheral blood were co-incubated with selected and unselected spermatozoa, and the interaction between PMN and spermatozoa was analyzed. In conclusion, SLC of fresh donkey semen increases the proportion of functionally intact spermatozoa and appears to remove the SP proteins that inhibit sperm-PMN binding, thus increasing sperm phagocytosis by PMN.

**Abstract:**

This study sought to determine whether single layer centrifugation (SLC) of fresh donkey semen with Equicoll has any impact on sperm quality parameters and on the modulation of endometrial reaction following semen deposition using an in vitro model. Seventeen ejaculates from five jackasses were obtained using an artificial vagina and diluted in a skim-milk extender. Samples were either selected through SLC (Equicoll) or non-treated (control). Two experiments were performed. The first one consisted of incubating selected or non-selected spermatozoa at 38 °C for 180 min. Integrity and lipid disorder of sperm plasma membrane, mitochondrial membrane potential, and intracellular levels of calcium and reactive oxygen species were evaluated at 0, 60, 120, and 180 min. In the second experiment, polymorphonuclear neutrophils (PMN) isolated from jennies blood were mixed with selected and unselected spermatozoa. Interaction between spermatozoa and PMN was evaluated after 0, 60, 120, and 180 min of co-incubation at 38 °C. SLC-selection increased the proportions of spermatozoa with an intact plasma membrane and low lipid disorder, of spermatozoa with high mitochondrial membrane potential and with high calcium levels, and of progressively motile spermatozoa. In addition, selection through SLC augmented the proportion of phagocytosed spermatozoa, which supported the modulating role of seminal plasma proteins on sperm-PMN interaction. In conclusion, SLC of fresh donkey semen increases the proportions of functionally intact and motile spermatozoa, and appears to remove the seminal plasma proteins that inhibit sperm-PMN binding.

## 1. Introduction

Proper management of breeding and genetic diversity in equine species requires the use of assisted reproduction technologies (ART), which includes artificial insemination (AI), embryo transfer (ET), intracytoplasmic sperm injection (ICSI), and cryopreservation [1,2]. It has been reported that when sperm are selected, fertility outcomes following ART increase [3,4]. This selection is usually based on sperm motility, morphology, and the integrities of plasma membrane, acrosome, and DNA. In addition, sperm selection allows for the removal of seminal plasma, non-viable sperm, pathogens, and debris particles [5]. While seminal plasma has been described to have a detrimental effect on sperm motility and viability during storage, the presence of non-viable, morphologically abnormal spermatozoa may be a source of reactive oxygen species (ROS), which can also be detrimental to sperm survival during storage [6]. 

Several sperm preparation techniques have been designed to separate male gametes from seminal plasma and to select spermatozoa with better quality. Because simple sperm washing does not remove all seminal plasma components [7], the other two alternatives, i.e., sperm migration and colloid centrifugation, are the most used in equine practice [8]. Sperm migration through swim-up is based on sperm motility and yields low recovery rates [7]. In contrast, centrifugation with colloids uses silane-coated silica particles in a species-specific formulation that allows the separation of heterogeneous sperm into sub-populations according to their density. Not only does this technique select sperm on the basis of their motility, but also on their normal morphology, and plasma membrane and chromatin integrities [8].

Single layer centrifugation (SLC) is a simplification of the density gradient centrifugation as only one layer of colloid is employed [9]. Previous studies have demonstrated that, following SLC, spermatozoa show higher motility, less DNA fragmentation, and higher pregnancy rates in subfertile stallions [10,11]. With respect to jackasses, SLC using Equicoll improves sperm quality parameters, such as motility, viability, and morphology, after 24 h of cooled storage [12], and after thawing of cryopreserved doses [13]. To the best of our knowledge, however, no study with fresh donkey semen has been performed nor has any evaluated the effects of SLC with Equicoll on mitochondrial activity, and intracellular levels of ROS and calcium. Other studies in rams [14], pigs [15], bulls [16], and brown bears [17,18] have demonstrated that centrifugation with SLC increases the proportions of spermatozoa with an intact plasma membrane and acrosome, high mitochondrial activity, an intact DNA, and progressive motility, and has also been found to increase sperm cryotolerance when performed before cryopreservation [19,20]. 

While elimination of seminal plasma is beneficial for sperm cryopreservation [21] and improves the stability of plasma membrane in fresh and cooled-stored stallion spermatozoa [22], one should bear in mind that several seminal plasma factors are involved in the regulation of the inflammatory response within the female reproductive tract. In addition, studies conducted in other species, such as the pig, showed that SLC removes seminal plasma components, including porcine spermadhesins PSP-I and PSP-II, and some cholesterol molecules from sperm plasma membrane [23]. Remarkably, the presence of seminal plasma has been shown to reduce chemotaxis, sperm-neutrophil binding, phagocytosis, and the formation of DNA-based neutrophil extracellular traps (NETs) [24]. In the donkey, in vitro models suggest that seminal plasma can suppress sperm-PMN attachment [25,26] and that, when added to frozen/thawed sperm, downregulates the expression of *COX2* in endometrial cells, which leads PMN chemotaxis to reduce [27]. 

Against this background, we hypothesized that SLC of jackass semen with Equicoll could increase the proportions of donkey spermatozoa exhibiting high motility, an intact and functional plasma membrane and high mitochondrial activity, and modify sperm-PMN binding. The effects of SLC on the proportions of spermatozoa with high levels of intracellular calcium and reactive oxygen species were also investigated. In addition, we tested the effects of SLC with Equicoll on donkey sperm not only after centrifugation, but also after 60, 120, and 180 min of incubation at 38 °C. The rationale of testing SLC-effects over this incubation period is related to the time that sperm are deemed to reside within the uterus (3 h).

## 2. Materials and Methods 

### 2.1. Animals

Semen samples (N = 17) were collected from five Catalonian jackasses, whose age ranged from 3 to 11 years, that were in good health and shown to be fertile. Jackasses underwent a regular semen collection, every other day during the week, and were housed in individual paddocks at the Equine Reproduction Service, Autonomous University of Barcelona (Bellaterra, Cerdanyola del Vallès, Spain), which is an EU-approved equine semen collection center (ES09RS01E). 

Polymorphonuclear neutrophils were isolated from the peripheral blood of three Catalonian jennies as described previously [26]. These animals, which were also in good health and of proven fertility, were housed in a big paddock at the Equine Reproduction Service, Autonomous University of Barcelona.

Animals were handled following the European Union Directive 2010/63/EU for animal experiments and the Animal Welfare Law from the Regional Government of Catalonia (Spain). In addition, this study was approved by the Ethics Committee, Autonomous University of Barcelona (Code: CEEAH 1424).

### 2.2. Experimental Design

This study consisted of two separate experiments. The goal of the first experiment was to determine the effects of SLC with Equicoll on sperm function parameters, such as motility, plasma membrane integrity, membrane lipid disorder, intracellular calcium levels, intracellular ROS levels, and mitochondrial activity. The effects of SLC-selection on donkey sperm subpopulations were also considered. Although previous works evaluated the effects of SLC with Equicoll on donkey semen, they used cooled-stored and cryopreserved sperm [12,13]; in contrast, the current study used fresh semen. Ejaculates (N = 10) were collected through a Hannover artificial vagina (Minitüb GmbH, Tiefenbach, Germany) with an in-line nylon mesh filter to remove gel and debris. Immediately after collection, gel-free semen was extended at a ratio of five volumes of skim milk extender (4.9% glucose, 2.4% skim milk, 100 mL double distilled water), previously warmed at 38 °C, and one volume of semen. After assessing sperm concentration, viability, morphology, and motility, each ejaculate was divided into two fractions. The first one was utilized as a control and was directly adjusted to a final concentration of 25 × 10^6^ sperm/mL. The second fraction was centrifuged through a single layer of a silane-coated silica-based colloid formulation (Equicoll, SLU, Sweden; formerly known as Androcoll-E) according to the protocol for small centrifuge tubes described by Morrell et al. [28]. Briefly, 1.5 mL of extended semen containing up to 100 × 10^6^ sperm/mL was pipetted on top of 4 mL Equicoll in a 15-mL tube. After centrifugation at 300× *g* and room temperature for 20 min, the supernatant and most of the colloid was discarded and the sperm pellet was transferred to a clean centrifuge tube containing 0.5 mL of the same skim milk extender. Sperm concentration was evaluated and subsequently adjusted to a final concentration of 25 × 10^6^ sperm/mL. Aliquots were incubated at 38 °C under aerobic conditions for 3 h. Sperm motility and other functional parameters evaluated with flow cytometry were determined immediately after SLC-selection (0 min), and after 60 min, 120 min, and 180 min of incubation at 38 °C.

The second experiment aimed to determine the effects of SLC with Equicoll on the interaction of donkey sperm with polymorphonuclear neutrophils (PMN). With this purpose, each ejaculate (N = 7) was split into two fractions. One fraction was used as a control and its sperm concentration was adjusted to 500 × 10^6^ sperm/mL. The other fraction was centrifuged through a single layer of a silane-coated silica-based colloid formulation (Equicoll) as described for Experiment 1. After centrifugation, sperm concentration was evaluated and subsequently adjusted to a final concentration of 500 × 10^6^ sperm/mL. Treatments consisted of in vitro co-incubation of sperm cells with PMN-rich samples (1:1; v:v) in a water bath at 38 °C for 3 h, as previously described by Miró et al. [26]. Briefly, peripheral blood neutrophils were isolated from healthy jennies. Following incubation in a water bath at 38 °C for 30 min, heparinized blood was subjected to centrifugation at 400× *g* and 4 °C for 5 min prior to removing plasma. The buffy coat was mixed with an isotonic saline solution (PBS) and again centrifuged at 400× *g* and 4 °C for 5 min. The PMN-rich buffy coat was collected and re-suspended in PBS to a final concentration of 100 × 10^6^ PMN/mL. All samples (selected and non-selected) were examined for sperm phagocytosis immediately after SLC-selection (0 min), and after 60 min, 120 min, and 180 min of incubation at 38 °C. 

### 2.3. Flow Cytometry 

Sperm quality parameters were determined using a Cell Laboratory Quanta SC^TM^ flow cytometer (Beckman Coulter; Fullerton, CA, USA). The following sperm parameters were evaluated: plasma membrane integrity (viability), membrane lipid disorder, acrosome integrity, mitochondrial activity (mitochondrial membrane potential), and intracellular levels of calcium (Ca^2+^), superoxides (O_2_*^−●^*) and peroxides (H_2_O_2_). Prior to any staining, each sample was diluted with HEPES buffered saline solution (10 mmol/L HEPES (4-(2-hydroxyethyl)-1-piperazineethanesulfonic acid); 150 mmol/L NaCl, 10% BSA; pH = 7.4) to a final concentration of 1 × 10^6^ spermatozoa/mL. Samples were subsequently incubated with the corresponding fluorochromes under dark conditions. After fluorochrome staining, an argon ion laser was used to excite samples at 488 nm (22 mW). Spermatozoa were selected on the basis of electronic volume (EV) and side scatter (SS) and the sheath flow rate was set at 4.17 µL/min. Based on EV/SS dot-plots, sperm cells were selected and the other events, including subcellular debris (particle diameter <7 µm) and cell aggregates (particle diameter >12 µm), were gated out. The flow cytometer was calibrated every day using 10-μm flow-check fluorospheres (Beckman Coulter), positioning the bead size (10 μm) on the EV channel (EV = 200). 

Up to 10,000 events were analyzed per replicate and a total of three independent replicates per sample were evaluated. FL1 detector (BP, band pass: 525 nm; DRLP, dichroic long pass: 550 nm) was used for monitoring the green fluorescence emitted by SYBR14, YO-PRO-1 (1-(4-[3-methyl-2,3-dihydro-(benzo-1,3-oxazole)-2-methylidene]-quinolinium)-3trimethylammonium propane diodide), JC1_mon_ (5,5′,6,6′-tetrachloro-1,1′3,3′tetraethyl-benzimidazolylcarbocyanine iodide monomers), H_2_DFCDA (2′,7′-dichlorodihydrofluorescein diacetate), and Fluo3-AM (acetoxymethyl, ester form) fluorochromes. FL2 detector (BP: 575 nm; DRLP: 600 nm) was used for monitoring the orange fluorescence emitted by JC1_agg_. FL3 detector (LP, long pass: 670 nm) was used for monitoring the red fluorescence emitted by HE (hydroethidine), PI (propidium iodide), M540 (merocyanine 540), and Rhod5.

Post-acquisition analyses were conducted using the Cell Lab Quanta^®^ SC MPL Analysis Software (version 1.0; Beckman Coulter; Fullerton, CA, USA). Data were corrected by subtracting the percentage of debris particles found in SYBR14/PI staining from the percentages of particles appearing at the lower left quadrant in the other tests [29]. The proportions of the other sperm populations were normalized.

#### 2.3.1. Evaluation of Sperm Membrane Integrity (SYBR14/PI)

Sperm viability was assessed using a combination of two fluorochromes (SYBR14 and propidium iodide, PI) available from a commercial kit (Live/Dead Sperm Viability kit, Molecular Probes, ThermoFisher Scientific; Waltham, MA, USA). After adjusting cell concentration to 1 × 10^6^ sperm/mL, spermatozoa were stained with SYBR14 (final concentration: 100 nM) at 38 °C for 10 min. Following this, PI (final concentration: 12 µM) was added and samples were incubated at 38 °C for 5 min. The following three categories of spermatozoa were identified: (i) spermatozoa with an intact plasma membrane, stained in green (viable spermatozoa; SYBR14^+^/PI^−^); (ii) spermatozoa with an altered plasma membrane, stained in red (non-viable spermatozoa; SYBR14^−^/PI^+^); and (iii) spermatozoa with an altered plasma membrane, stained in both red and green (non-viable spermatozoa; SYBR14^+^/PI^+^). Non-DNA containing particles (debris particles; SYBR14^−^/PI^−^), which appeared at the lower left quadrant, were subtracted from the total number of events and, as aforementioned, were used to correct the other tests. The protocol included compensation of FL1-spill over into the FL3-channel (2.45%).

#### 2.3.2. Evaluation of Sperm Membrane Lipid Disorder (M540/YO-PRO-1)

Lipid disorder of plasma membrane was evaluated following the protocol set by Rathi et al. [30] with minor modifications [31], which is based on M540 and YO-PRO-1 fluorochromes (Molecular Probes, ThermoFisher Scientific). Briefly, samples were incubated with M540 (final concentration: 2.6 µM) and YO-PRO-1 (final concentration: 25 nM) at 38 °C for 10 min. A total of four sperm populations was identified in flow cytometry dot-plots: (1) viable spermatozoa with low membrane lipid disorder (M540^−^/YO-PRO-1^−^); (2) viable spermatozoa with high membrane lipid disorder (M540^+^/YO-PRO-1^−^); (3) non-viable spermatozoa with low membrane lipid disorder (M540^−^/YO-PRO-1^+^); and (4) non-viable spermatozoa with high membrane lipid disorder (M540^+^/YO-PRO-1^+^). Correction was performed following the procedure described previously and data were not compensated.

#### 2.3.3. Evaluation of Mitochondrial Membrane Potential (JC1)

Assessment of mitochondrial membrane potential (MMP) was conducted using JC1-staining (Molecular Probes, ThermoFisher Scientific), following the protocol set by Ortega-Ferrusola et al. [32]. With this purpose, samples were incubated with JC1 (final concentration: 0.3 µM) at 38 °C for 30 min. When MMP is low, JC1 remains as monomers (JC1_mon_); when MMP is high, JC1 forms aggregates (JC1_agg_). Three different sperm populations were identified: (1) spermatozoa with green-stained mitochondria, which had low MMP (JC1_mon_); (2) spermatozoa with orange-stained mitochondria; and (3) spermatozoa with green- and orange-stained mitochondria. Populations 2 and 3 were considered as having high MMP (JC1_agg_). The protocol included compensation of FL1-spill over into the FL3-channel (68.50%).

#### 2.3.4. Evaluation of Intracellular Calcium Levels (Fluo3/PI and Rhod5/YO-PRO-1)

Two different co-staining protocols were used to determine intracellular calcium levels. Fluo3-AM is able to penetrate cell membranes and displays better affinity for the calcium present in the sperm mid-piece [33]. This test, which is combined with PI, was performed as described by Harrison et al. [34] and Kadirvel et al. [35]. Briefly, spermatozoa were incubated with Fluo3-AM (final concentration: 1 µM) and PI (final concentration: 12 µM) at 38 °C for 10 min. Four different sperm populations were identified: (1) viable spermatozoa with low levels of intracellular calcium (Fluo3^−^/PI^−^); (2) viable spermatozoa with high levels of intracellular calcium (Fluo3^+^/PI^−^); (3) non-viable spermatozoa with low levels of intracellular calcium (Fluo3^−^/PI^+^); and (4) non-viable spermatozoa with high levels of intracellular calcium (Fluo3^+^/PI^+^). The protocol included compensation of FL1-spill over into the FL3-channel (28.72%), and of FL3-spill over into the FL1-channel (2.45%).

Rhod5 has more affinity for the calcium residing in the sperm head than for that found in the mid-piece. This test was performed following the protocol set by Yeste et al. [33]. In brief, spermatozoa were incubated with Rhod-5N (final concentration: 5 µM) and YO-PRO-1 (final concentration: 25 nM) at 38 °C for 10 min. Four sperm populations were distinguished in flow cytometry dot-plots: (1) viable spermatozoa with low levels of intracellular calcium (Rhod5^−^/YO-PRO-1^−^); (2) viable spermatozoa with high levels of intracellular calcium (Rhod5^+^/YO-PRO-1^−^); (3) non-viable spermatozoa with low levels of intracellular calcium (Rhod5^−^/YO-PRO-1^+^); and (4) non-viable spermatozoa with high levels of intracellular calcium (Rhod5^+^/YO-PRO-1^+^). The protocol included compensation of FL3-spill over into the FL1-channel (3.16%).

#### 2.3.5. Evaluation of Intracellular Reactive Oxygen Species (ROS) Levels: H_2_O_2_ and O_2_^−●^ (H_2_DCFDA/PI and HE/YO-PRO-1)

The evaluation of intracellular levels of hydrogen peroxides (H_2_O_2_) was performed following the protocol set by Morrell et al. [36]. In brief, sperm samples were stained with H_2_DCFDA (final concentration: 140 µM) and PI (final concentration: 12 µM) and incubated at 25 °C for 30 min. H_2_DCFDA consists of a non-fluorescent probe that is able to penetrate plasma membranes. Upon intracellular oxidation, H_2_DCFDA is de-esterified and converted into highly green fluorescent 2′,7′-dichlorofluorescein (DCF^+^), which is detected through FL1. Flow cytometry dot-plots depicted four sperm populations: (1) viable spermatozoa with high levels of intracellular peroxides (DCF^+^/PI^−^); (2) viable spermatozoa with low levels of intracellular peroxides (DCF^−^/PI^−^); (3) non-viable spermatozoa with high levels of intracellular peroxides (DCF^+^/PI^+^); and (4) non-viable spermatozoa with low levels of intracellular peroxides (DCF^−^/PI^+^). The protocol included compensation of FL1-spill over into the FL3-channel (2.45%).

The determination of intracellular levels of superoxide (O_2_^−●^) radicals was performed through co-staining with hydroethidine (HE) and YO-PRO-1, following the protocol set by Guthrie and Welch [37]. Briefly, sperm samples were mixed with HE (final concentration: 4 µM) and YO-PRO-1 (final concentration: 25 nM) and incubated at 25 °C for 30 min. In the presence of O_2_^−●^, HE, which is known to pass across plasma membranes, is oxidized to ethidium (E^+^; detected by FL3). A total of four sperm populations were identified: (1) viable spermatozoa with high levels of intracellular superoxides (E^+^/YO-PRO-1^−^); (2) viable spermatozoa with low levels of intracellular superoxides (E^−^/YO-PRO-1^−^); (3) non-viable spermatozoa with high levels of intracellular superoxides (E^+^/YO-PRO-1^+^); and (4) non-viable spermatozoa with low levels of intracellular superoxides (E^−^/YO-PRO-1^+^). The protocol included compensation of FL1-spill over into the FL3-channel (5.06%).

### 2.4. Evaluation of Sperm Motility

Sperm motility and kinetic parameters were assessed using a computer-assisted sperm motility analysis (CASA) system (ISAS v. 1.0; Proiser S.L., Valencia, Spain) and an Olympus BX41 microscope (Olympus 20x 0.30 PLAN objective; Olympus Europe, Hamburg, Germany). With this purpose, 5 µL of each sample was placed into a Neubauer Chamber and then observed using a phase contrast microscope with a pre-warmed stage (38 °C). For each sample, three fields per drop and at least 1000 spermatozoa were assessed. The following variables were analyzed: percentages of total motile spermatozoa (TMOT), percentages of progressively motile spermatozoa (PMOT), straight line velocity (VSL), curvilinear velocity (VCL), average path velocity (VAP), linearity index (LIN), straightness index (STR), oscillation index (WOB), amplitude of lateral head displacement (ALH), and beat cross frequency (BCF) [38]. Cut-off values for a given sperm cell to be considered as motile or progressively motile were VAP ≥ 10 µm/s and STR ≥ 75%, respectively.

### 2.5. Evaluation of Sperm–PMN Binding

Smears were prepared on microscope slides for each treatment and stained with a modified Wright’s dye (Diff Quick^®^, Quimica Clinica Aplicada, Amposta, Spain). Phagocytosis was determined at 1000× magnification (Olympus Europe, Hamburg, Germany) under immersion oil, and was expressed as the percentage of PMN that ingested at least one spermatozoon [26]. The percentages of spermatozoa in contact with PMN were also counted. Two hundred PMN per slide were counted, and three technical replicates were made.

### 2.6. Statistical Analysis

Data obtained from the analysis of all sperm parameters were first tested for normality (Shapiro–Wilk test) and homogeneity of variances (Levene test). Following this, all variables except those related to the effects of SLC-selection upon sperm motile populations (i.e., % SYBR14^+^/PI^−^ spermatozoa, % M540^−^/YO-PRO-1^−^ spermatozoa, % spermatozoa with high MMP, JC1_agg_/JC1_mon_ ratios, % Fluo3^+^/viable spermatozoa, % Rhod5^+^/viable spermatozoa, % DCF^+^/viable spermatozoa, % E^+^/viable spermatozoa, % TMOT spermatozoa, % PMOT spermatozoa, VCL, VSL, VAP, LIN, STR, WOB, ALH, and BCF) were evaluated through a linear mixed model (intra-subjects factor: time of incubation; inter-subjects factor: control vs. SLC-Equicoll), followed by Sidak post-hoc test. 

In order to classify spermatozoa into separate motile subpopulations, the procedure described by Luna et al. [39] was conducted with minor modifications; this procedure combines Principal Component (PCA) and cluster analyses. First, a PCA with the individual kinematic parameters (VSL, VCL, VAP, LIN, STR, WOB, ALH, and BCF) obtained for each spermatozoon (CASA assessment) was run; data were sorted into PCA components and the matrix was rotated using the Varimax approach and Kaiser standardization. Thereafter, cluster analyses were conducted through a two-way procedure based on Schwarz’s Bayesian Criterion and log-likelihood distance using the individual regression scores of each PCA component. Four separate motile sperm subpopulations were identified, and proportions of spermatozoa belonging to each subpopulation (SP1, SP2, SP3, or SP4) were subsequently calculated. The effects of SLC-selection upon sperm motile populations were evaluated through a Scheirer–Ray–Hare ranked ANOVA followed by Mann–Whitney test for pair-wise comparisons. Proportions of SP1, SP2, SP3, and SP4 spermatozoa were considered as dependent variables.

Results are shown as mean ± standard error of the mean (SEM); the minimal level of significance was set at *p* ≤ 0.05 in all cases. 

## 3. Results

### 3.1. Integrity and Lipid Disorder of Sperm Plasma Membrane

**Figure 1:**
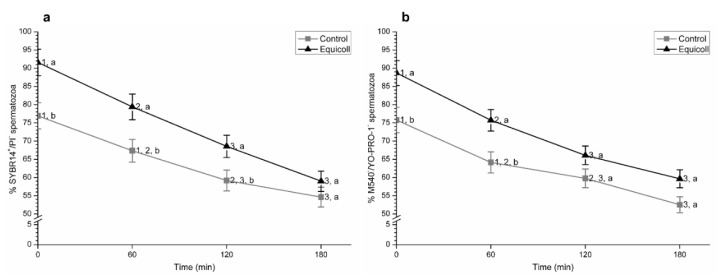
Percentages of (**a**) membrane-intact spermatozoa (SYBR14^+^/PI^−^) and (**b**) viable spermatozoa with low membrane lipid disorder (M540^−^/YO-PRO-1^−^) in control (non-selected samples) and SLC-selected samples (Equicoll). Different superscript letters (a, b) indicate significant differences (*p* ≤ 0.05) between treatments within a given time point, and different superscript numbers (1–3) mean significant differences between time points within the control or samples selected with Equicoll. Data are shown as mean ± SEM for 10 separate experiments. SLC: single layer centrifugation.

Figure 1a shows (mean ± SEM) the proportions of viable spermatozoa (i.e., spermatozoa with an intact plasma membrane; SYBR14^+^/PI^−^) in the control and samples selected through SLC with Equicoll. Incubation led to a significant (*p* < 0.05) decrease in the proportions of viable spermatozoa in both the control and SLC-selected samples. However, proportions of viable spermatozoa were significantly (*p* < 0.05) higher in samples selected through SLC than in their control counterparts, immediately after selection (0 min) and after 60 min and 120 min of incubation at 38 °C. At 180 min of incubation, no significant differences between treatments were observed. 

Proportions of viable spermatozoa with low membrane lipid disorder (M540^−^/YO-PRO-1^−^; mean ± SEM) also decreased (*p* < 0.05) over incubation time (Figure 1b). Again, these percentages were significantly (*p* < 0.05) higher in SLC-selected samples than in the control immediately after centrifugation (0 min) and after 60 min of incubation at 38 °C. No significant differences between the control and SLC-selected samples were observed after 120 min and 180 min of incubation.

### 3.2. Mitochondrial Membrane Potential

**Figure 2:**
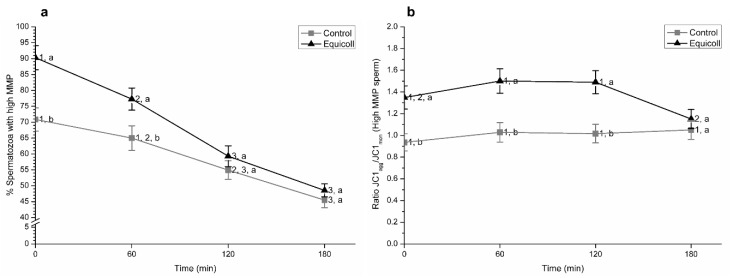
(**a**) Percentages of spermatozoa with high mitochondrial membrane potential (MMP) and (**b**) ratios between JC1_agg_ and JC1_mon_ of the sperm population with high MMP in control (non-selected samples) and SLC-selected samples (Equicoll). Different superscript letters (a, b) indicate significant differences (*p* ≤ 0.05) between treatments within a given time point, and different superscript numbers (1–3) mean significant differences between time points within the control or samples selected with Equicoll. Data are shown as mean ± SEM for 10 separate experiments. SLC: single layer centrifugation.

Incubation at 38 °C led to a significant (*p* < 0.05) decrease in the proportions of spermatozoa with high MMP (Figure 2a; mean ± SEM). Upon selection (0 min) and after 60 min of incubation at 38 °C, proportions of spermatozoa with high MMP were significantly (*p* < 0.05) higher in samples selected through SLC than in their control counterparts. No significant differences between the control and SLC-selected samples were observed after 120 min and 180 min of incubation at 38 °C. In addition, JC1_agg_/JC1_mon_ ratios in the sperm population with high MMP were significantly (*p* < 0.05) lower in the control than in SLC-selected samples at 0 min, and after 60 and 120 min of incubation at 38 °C (Figure 2b; mean ± SEM). 

### 3.3. Intracellular Calcium Levels

**Figure 3:**
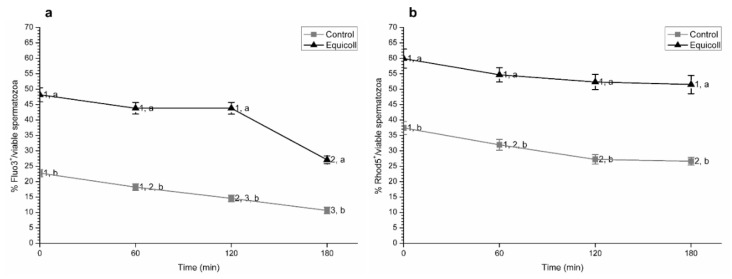
Proportions of viable spermatozoa with high intracellular calcium levels evaluated with two separate fluorochromes: Fluo3 (**a**) and Rhod5 (**b**) in control (non-selected samples) and SLC-selected samples (Equicoll). Data are given considering the viable sperm population. Different superscript letters (a, b) indicate significant differences (*p* ≤ 0.05) between treatments within a given time point, and different superscript numbers (1–3) indicate significant differences between time points within the control or samples selected with Equicoll. Results are shown as mean ± SEM for 10 separate experiments. SLC: single layer centrifugation.

As shown in Figure 3a (mean ± SEM), proportions of spermatozoa with high levels of intracellular calcium calculated over the total number of viable spermatozoa and evaluated through both Fluo3 and Rhod5 fluorochromes were significantly (*p* < 0.05) higher in SLC-samples than in the control, not only immediately after selection (0 min) but also after 60 min, 120 min, and 180 min of incubation at 38 °C. While proportions of spermatozoa with high levels of intracellular calcium calculated over the total number of viable spermatozoa and evaluated through Fluo3/PI staining significantly (*p* < 0.05) decreased in SLC-selected samples after 180 min of incubation at 38 °C, this effect was not observed when the Rhod5/YO-PRO-1 test was used (mean ± SEM; Figure 3b).

### 3.4. Intracellular Levels of Peroxides and Superoxides

**Figure 4:**
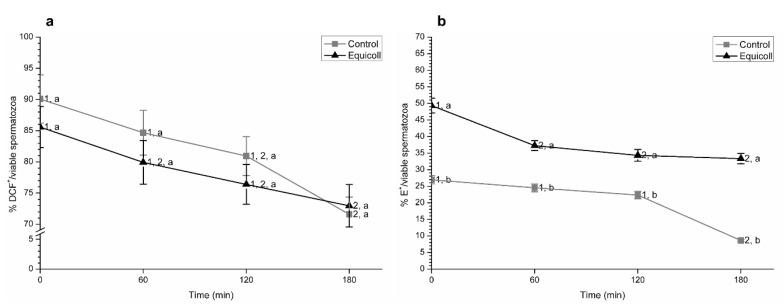
Proportions of viable spermatozoa with high intracellular levels of peroxides (DCF^+^; **a**) and superoxides (E^+^; **b**) in control (non-selected samples) and SLC-selected samples (Equicoll). Data are given considering the viable sperm population. Different superscript letters (a, b) indicate significant differences (*p* ≤ 0.05) between treatments within a given time point, and different superscript numbers (1–3) mean significant differences between time points within the control or samples selected with Equicoll. Results are shown as mean ± SEM for 10 separate experiments. SLC: single layer centrifugation.

Figure 4 shows, as mean ± SEM, the proportions of spermatozoa with high levels of peroxides and superoxides over the number of viable spermatozoa. While incubation at 38 °C significantly (*p* < 0.05) decreased the proportions of spermatozoa with high levels of intracellular peroxides over the total number of viable spermatozoa (DCF^+^/viable spermatozoa), no significant differences between the control and SLC-selected samples were observed (Figure 4a). In contrast, not only did incubation at 38 °C induce a significant (*p* < 0.05) decrease in the proportions of spermatozoa with high levels of superoxides over the total number of spermatozoa (E^+^/viable spermatozoa), but SLC-selected samples showed significantly (*p* < 0.05) higher values of this parameter than their control counterparts (Figure 4b).

### 3.5. Sperm Motility

**Figure 5:**
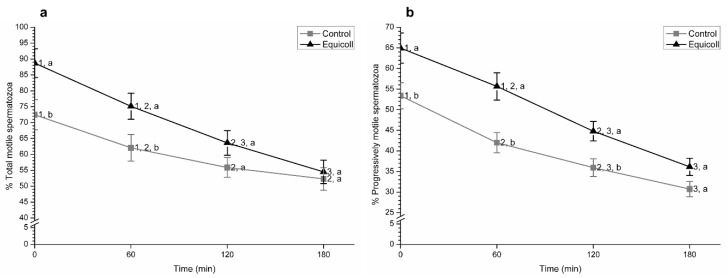
Proportions of (**a**) total and (**b**) progressively motile spermatozoa in control (non-selected samples) and SLC-selected samples (Equicoll). Different superscript letters (a, b) indicate significant differences (*p* ≤ 0.05) between the control and SLC-selected samples, and different superscript numbers (1–3) mean significant (*p* ≤ 0.05) differences between time points within the control or samples selected with Equicoll. Data are shown as mean ± SEM for 10 separate experiments. SLC: single layer centrifugation.

Proportions of total and progressively motile spermatozoa after SLC-selection and incubation at 38 °C are shown, as mean ± SEM, in Figure 5. Proportions of total and progressively motile spermatozoa were significantly (*p* < 0.05) higher in SLC-samples than in the control at 0 and 60 min. After 120 min of incubation at 38 °C, progressive but not total motility was significantly (*p* < 0.05) higher in SLC-selected samples than in the control. 

Sperm kinematics parameters are shown, as mean ± SEM, in Table 1. After 120 min of incubation at 38 °C, samples selected with SLC showed significantly (*p* < 0.01) lower values of VCL than the control. After 60 min, 120 min, and 180 min of incubation, VSL and VAP were significantly (*p* < 0.01) lower in SLC-selected samples than in the control. At 180 min, significantly (*p* < 0.05) lower values of WOB were observed in samples selected through SLC than in their control counterparts.

### 3.6. Motile Sperm Subpopulations

A two-step clustering procedure based on log-likelihood distance and the Schwarz Bayesian criterion was run with 24,399 motile spermatozoa. Four sperm subpopulations (SP1, SP2, SP3, and SP4) were identified. SP1 and SP2 exhibited the highest average path velocity (VAP), whereas SP3 was characterized by moderate VAP, and SP4 showed the lowest VAP (Table 2; mean ± SEM).

**Figure 6:**
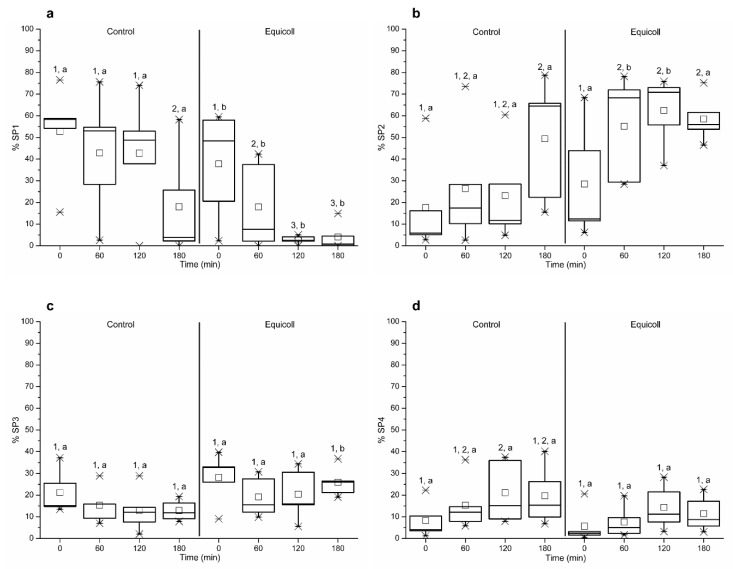
Proportions of spermatozoa belonging to one of each sperm subpopulations ((**a**), SP1; (**b**), SP2; (**c**), SP3; (**d**), SP4) in control (non-selected samples) and SLC-selected samples (Equicoll). Different superscript letters (a, b) indicate significant differences (*p* ≤ 0.05) between treatments within a given time point, and different superscript numbers (1–3) mean significant differences between time points within the control or samples selected with Equicoll. Data are shown as box-whisker plots for 10 separate experiments. SLC: single layer centrifugation. SP1: subpopulation 1; SP2: subpopulation 2; SP3: subpopulation 3; SP4: subpopulation 4.

Immediately after centrifugation with Equicoll (0 min) and after 60, 120, and 180 min of incubation at 38 °C, proportions of motile spermatozoa belonging to SP1 were significantly (*p* < 0.05) lower in SLC-samples than in the control (Figure 6a). The proportions of motile spermatozoa belonging to SP2 were significantly (*p* < 0.05) lower in the control than in SLC-selected samples (Figure 6b). With regard to SP3, the proportions of spermatozoa belonging to that population were significantly (*p* < 0.05) higher in SLC-selected samples than in the control after 180 min of incubation (Figure 6c). Finally, no significant differences in the proportions of sperm belonging to SP4 were observed between the control and SLC-selected samples either at 0 min or over the incubation period at 38 °C (60, 120, and 180 min; Figure 6d).

### 3.7. Sperm-PMN Interaction

**Figure 7:**
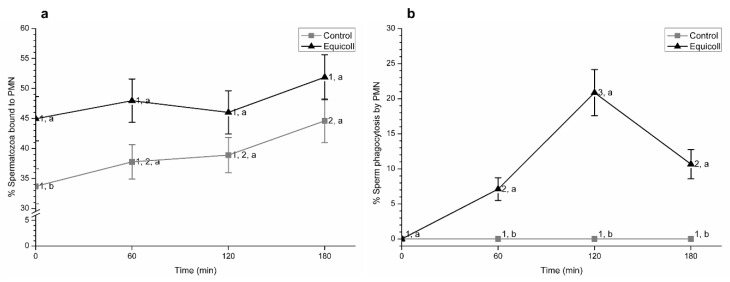
Percentages of (**a**) spermatozoa bound to PMN and (**b**) sperm phagocytosed by PMN in control (non-selected samples) and SLC-selected samples (Equicoll). Different superscript letters (a, b) indicate significant differences (*p* ≤ 0.05) between treatments within a given time point, and different superscript numbers (1, 2) mean significant differences between time points within the control or samples selected with Equicoll. Data are shown as mean ± SEM for 7 separate experiments. SLC: single layer centrifugation. PMN: Polymorphonuclear neutrophils.

At 0 min, proportions of sperm bound to PMN were significantly (*p* < 0.05) higher in SLC-selected samples than in the control. In contrast, no significant differences between control and SLC-selected samples were observed after 60, 120, or 180 min of incubation (Figure 7a; mean ± SEM). Moreover, and as shown in Figure 7b (mean ± SEM), percentages of spermatozoa phagocytosed by PMN were significantly (*p* < 0.05) higher in SLC-selected samples than in the control after 60, 120, and 180 min of incubation at 38 °C. 

## 4. Discussion

Pregnancy rates after AI are affected by sperm quality and by the ability of the female reproductive tract to support an embryo. Consequently, selection of high-quality sperm represents a challenge in equine assisted reproduction. Previous studies have already shown that SLC with cooled-stored and frozen/thawed semen significantly improves sperm quality parameters in both stallions [11] and jackasses [12]. On the other hand, one should note that using a species-specific formulated colloid is highly advisable, as the specific particularities of each species require the design of concrete formulations. In the current study, we observed that fresh donkey semen samples previously centrifuged with SLC (Equicoll) showed higher proportions of spermatozoa with an intact plasma membrane (viable spermatozoa), low membrane lipid disorder, high intracellular calcium (which was evaluated with two separate fluorochromes [33,34]), high superoxide levels, and high MMP, and of progressively and total motile spermatozoa than the control. In contrast, after centrifugation with SLC, spermatozoa showed lower VSL, VCL, and VAP, and the proportions of spermatozoa belonging to SP1, which was the motile sperm subpopulation that exhibited the highest VAP, were also significantly lower than the control. Furthermore, the removal of seminal plasma components due to centrifugation with SLC led to an increase in the percentages of sperm phagocytosed by PMN after 60, 120, and 180 min of incubation, and in the percentages of spermatozoa bound to PMN, although in this case significant differences were only observed at 0 min. 

Overall, our data support that SLC selects a sperm functional population, which exhibits high plasma membrane integrity. This increase in plasma membrane integrity is not only apparent from the analysis of SYBR14/PI staining but also from that of M540/YO-PRO-1. However, the higher plasma membrane integrity and lower membrane lipid disorder of SLC-selected spermatozoa compared to the control were statistically significant immediately after centrifugation and after 60 min of incubation at 38 °C but not later, which indicates that further incubation at this temperature abolishes these differences. In this context, it is worth mentioning that the 3-h period was set to mimic the time deemed for sperm to reside within the uterus. However, this post-selection incubation time was not included in previous studies, which made our results difficult to compare. Moreover, most of the previous works investigating how selection with SLC affects sperm quality were focused on stored semen (cooled and frozen). In donkeys, Ortiz et al. obtained significantly higher values of sperm quality parameters with colloid centrifugation using Equicoll after 24 h of cooled storage [12] and after freezing/thawing [13]. It is worth highlighting that sperm samples included in this study were initially within the standard values for donkey semen [40]. In this regard, even if SLC-selected spermatozoa are the ones exhibiting the highest quality, it is difficult to observe much apparent differences between the control and SLC-selected samples when the raw semen is of good quality. Under this perspective, our results are in agreement with those obtained by Ortiz et al. who observed that the use of SLC-selection to improve post-thaw sperm quality yielded better results when ejaculates with poor freezability were used [41]. Be that as it may, further research comparing the effects of SLC-selection on groups of fresh ejaculates with high and low sperm quality is warranted. 

As aforementioned, in this work, we found a significant improvement in different sperm function parameters. In agreement with our data, centrifugation with species-specific designed colloids gives similar results. In horses, centrifugation of cooled-stored semen with Equicoll has been reported to increase the proportions of the most functional spermatozoa [42,43]. Despite not being evaluated in our study, the aforementioned research also demonstrated that SLC with Equicoll removes much of the bacterial load present in their ejaculates [42,43] and increases pregnancy rates after AI [44,45]. In rams, Šterbenc et al. [14] observed that centrifugation of frozen/thawed semen with a species-specific colloid (Androcoll-O) increases the proportions of spermatozoa with an intact plasma membrane and DNA, and of motile spermatozoa. In pigs, SLC with Porcicoll (formerly known as Androcoll-P) has been found to enhance the quality of frozen/thawed sperm, in terms of viability, mitochondrial activity and intracellular levels of ROS [15]. Also in pigs, centrifugation of semen with SLC with Porcicoll prior to cryopreservation increases the cryotolerance and fertilizing ability of frozen/thawed sperm [19,20]. Even in brown bears, centrifugation with SLC has also been reported to improve the proportion of spermatozoa with intact plasma membrane and acrosome [17,18]. In bulls, centrifugation through SLC with a species-specific colloid (Androcoll-B/Bovicoll) also results in an increase in the proportions of spermatozoa with high mitochondrial membrane potential and high relative levels of peroxides [16], and in some kinematic parameters, but has no impact upon total and progressive motility of cooled-stored semen [46]. 

Another novel and interesting finding of this study is related to the evaluation of the percentages of sperm bound to PMN and those of sperm phagocytosed by PMN. We observed that, immediately after SLC-selection (0 min), the percentages of spermatozoa bound to PMN were higher in samples washed through SLC than in the control. In addition, the percentages of sperm phagocytozed by PMN were significantly higher in washed SLC-samples than in the control after 60, 120, and 180 min of incubation. As aforementioned, not only is centrifugation through SLC expected to select the sperm population with higher sperm quality and fertilizing ability, but it also removes seminal plasma components [23,47]. This means that donkey spermatozoa, especially when seminal plasma components are removed, are very susceptible to bind to PMN and, in some cases, be phagocytozed. In this context, it is worth mentioning that while sperm separation methods seek to mimic in vitro the natural selection occurring within the female reproductive tract [48], they also entail the removal of seminal plasma, which plays a crucial role in the response of the endometrium [26]. Upon ejaculation, specific components of seminal plasma, notably proteins such as spermadhesins, are adsorbed onto the sperm surface [49]. These sperm coating components confer important properties to the plasma membrane and prevent premature sperm capacitation [50]. Furthermore, previous studies have described that seminal plasma components are able to regulate the endometrial inflammatory reaction [25,51]. In effect, in the horse, the seminal protein CRISP3 has been found to inhibit the binding mechanism between viable spermatozoa and PMN involved in phagocytosis [52], improving fertility in mares [53]. In jennies, suppression of sperm-PMN binding by the presence of seminal plasma has also been identified [18], which would also be in agreement with the observed effect of seminal plasma in this study. We can thus hypothesize that sperm selection with Equicoll leads to the removal of seminal plasma components involved in the sperm protection from phagocytosis, since a previous study reported that the percentages of phagocytosis are low in the presence of seminal plasma [26]. In addition, one should also take into consideration that donkey semen has been reported to activate PMN and that PMN can create complexes known as neutrophil extracellular traps (NETosis) in the presence of spermatozoa [54,55,56]. NETosis is another type of programmed cell death that differs from apoptosis and is triggered by neutrophils (although monocytes and macrophages also show that ability, which is known as METosis). During NETosis, PMN undergo morphological changes, which include the disintegration of nuclear and granule membranes and the combination of nuclear, granular, and cytoplasmic components; the final result is the formation of traps made up of DNA and antimicrobial proteins. These extracellular traps allow capturing microbial agents and spermatozoa [57]. Therefore, further studies should elucidate whether the modifications of the sperm surface due to selection through SLC modify the ability of PMN to trigger NETosis, and determine the impact upon sperm fertilizing ability and endometrial reaction in the jenny.

## 5. Conclusions

We can conclude that previous centrifugation of donkey spermatozoa with SLC (Equicoll) likely selects the most functional spermatozoa, which are the ones that exhibit intact membranes, high mitochondrial membrane potential, and high intracellular calcium levels. In addition, selection with SLC increases the proportion of progressively and total motile spermatozoa. Finally, since selection of donkey sperm through SLC augments phagocytosis by PMN, further studies analyzing the effect of SLC-selection on sperm–PMN interaction, particularly NETosis, are much warranted.

## Figures and Tables

**Table 1:** Sperm kinematic parameters (as mean ± SEM) in control (non-selected samples) and SLC-selected samples (Equicoll).

Kinematic Parameters	0 min		60 min		120 min		180 min	
	Control	Equicoll	Control	Equicoll	Control	Equicoll	Control	Equicoll
VCL	155.0 ± 7.8 ^a^	138.4 ± 9.3 ^a^	143.4 ± 12.2 ^a^	102.5 ± 11.2 ^a^	136.3 ± 14.6 ^a^	84.8 ± 5.4 ^b^	108.5 ± 10.7 ^a^	67.7 ± 4.7 ^a^
VSL	115.3 ± 6.0 ^a^	96.8 ± 6.9 ^a^	112.4 ± 5.3 ^a^	80.6 ± 6.0 ^b^	109.1 ± 9.0 ^a^	68.5 ± 4.2 ^b^	106.9 ± 3.8 ^a^	53.9 ± 4.2 ^b^
VAP	141.6 ± 5.7 ^a^	121.9 ± 7.6 ^a^	130.0 ± 9.5 ^a^	91.9 ± 9.2 ^b^	124.7 ± 12.0 ^a^	76.3 ± 5.2 ^b^	100.1 ± 9.1 ^a^	58.9 ± 4.5 ^b^
LIN	74.7 ± 3.4 ^a^	70.2 ± 2.3 ^a^	79.6 ± 3.8 ^a^	79.8 ± 3.1 ^a^	81.6 ± 4.1 ^a^	81.0 ± 1.2 ^a^	84.5 ± 2.3 ^a^	79.5 ± 1.2 ^a^
STR	81.4 ± 2.6 ^a^	79.4 ± 2.5 ^a^	87.2 ± 3.0 ^a^	88.6 ± 2.5 ^a^	88.4 ± 3.0 ^a^	90.1 ± 1.9 ^a^	91.3 ± 1.6 ^a^	91.5 ± 0.8 ^a^
WOP	91.6 ± 1.7 ^a^	88.2 ± 1.0 ^a^	91.1 ± 1.6 ^a^	90.0 ± 1.1 ^a^	92.2 ± 1.6 ^a^	89.8 ± 0.7 ^a^	92.4 ± 1.4 ^a^	86.8 ± 1.2 ^b^
ALH	3.2 ± 0.4 ^a^	3.1 ± 0.3 ^a^	3.0 ± 0.4 ^a^	2.4 ± 0.2 ^a^	3.0 ± 0.4 ^a^	2.1 ± 0.1 ^a^	2.5 ± 0.3 ^a^	2.1 ± 0.1 ^a^
BCF	9.5 ± 0.8 ^a^	10.1 ± 1.1 ^a^	10.0 ± 10.9 ^a^	9.9 ± 0.6 ^a^	10.1 ± 1.0 ^a^	9.1 ± 0.2 ^a^	8.7 ± 0.1 ^a^	9.7 ± 0.3 ^a^

Different superscript letters (a, b) indicate significant differences (*p* ≤ 0.05) between treatments at 0, 60, 120, and 180 min. Data are shown as mean ± SEM for 10 separate experiments. VCL: sperm curvilinear velocity (µm/s); VSL: sperm linear velocity (µm/s); VAP: mean velocity (µm/s); LIN: linear coefficient (%); STR: straightness coefficient (%); WOB: wobble coefficient (%); ALH: mean lateral head displacement (µm); BCF: frequency of head displacement (Hz). SLC: single layer centrifugation.

**Table 2:** Kinematic parameters (mean ± SEM) of each motile sperm population.

Kinematic Parameters	SP1	SP2	SP3	SP4
*n*	8422	8133	6152	1692
VCL	165.8 ± 0.2	103.8 ± 0.3	120.1 ± 0.1	0.6 ± 0.1
VSL	132.8 ± 0.3	90.7 ± 0.3	46.4 ± 0.3	2.0 ± 0.1
VAP	150.5 ± 0.2	97.5 ± 0.3	94.4 ± 0.6	0.3 ± 0.0
LIN	80.2 ± 0.1	87.3 ± 0.1	40.6 ± 0.2	0.3 ± 0.1
STR	88.2 ± 0.1	93.2 ± 0.1	53.2 ± 0.3	0.67 ± 0.1
WOB	90.6 ± 0.1	93.4 ± 0.1	767.0 ± 0.1	1.0 ± 0.2
ALH	3.6 ± 0.0	2.1 ± 0.0	3.6 ± 0.0	0.1 ± 0.0
BCF	11.0 ± 0.0	8.4 ± 0.0	7.8 ± 0.0	0.1 ± 0.0

Abbreviations: SP1: subpopulation 1; SP2: subpopulation 2; SP3: subpopulation 3; SP4: subpopulation 4; VCL: sperm curvilinear velocity (µm/s); VSL: sperm linear velocity (µm/s); VAP: mean velocity (µm/s); LIN: linear coefficient (%); STR: straightness coefficient (%); WOB: wobble coefficient (%); ALH: mean lateral head displacement (µm); BCF: frequency of head displacement (Hz).
